# Organoids as brain tumour models: bridging the translational gap

**DOI:** 10.1242/dmm.052893

**Published:** 2026-09-01

**Authors:** Thomas Willott, Silvia Marino

**Affiliations:** Brain Tumour Research Centre of Excellence, Blizard Institute, 4 Newark Street, Queen Mary University of London, London E1 2AT, UK

**Keywords:** Brain organoids, Brain tumours, iPSCs, Stem cells, Glioblastoma, Medulloblastoma, Diffuse midline gliomas, Models

## Abstract

Advances in stem cell-derived brain organoids, enabled by the generation of human reprogrammed pluripotent stem cells, have significantly expanded the repertoire of models available for brain tumour research. These approaches have predominantly been applied to glioblastoma and medulloblastoma, leveraging brain region-specific cerebral/cortical or cerebellar organoids, respectively. Organoids have the potential to close the gap between current two-dimensional cell-based brain tumour models and patients, while helping to address the limitations of animal models. In this At a Glance article, we outline current stem cell-derived organoid-based brain tumour models and how they have expanded our understanding of paediatric and adult brain tumours, including their onset, heterogeneity and treatment. We also describe how these organoids are currently being refined toward more accurate, clinically relevant models and highlight future directions to improve their translatability.

## Teaser

**Summary:** This At a Glance article highlights recent advances and current limitations of region-specific stem cell-derived organoids to model relevant developmental and physiological contexts for brain tumour research.

**Figure:**
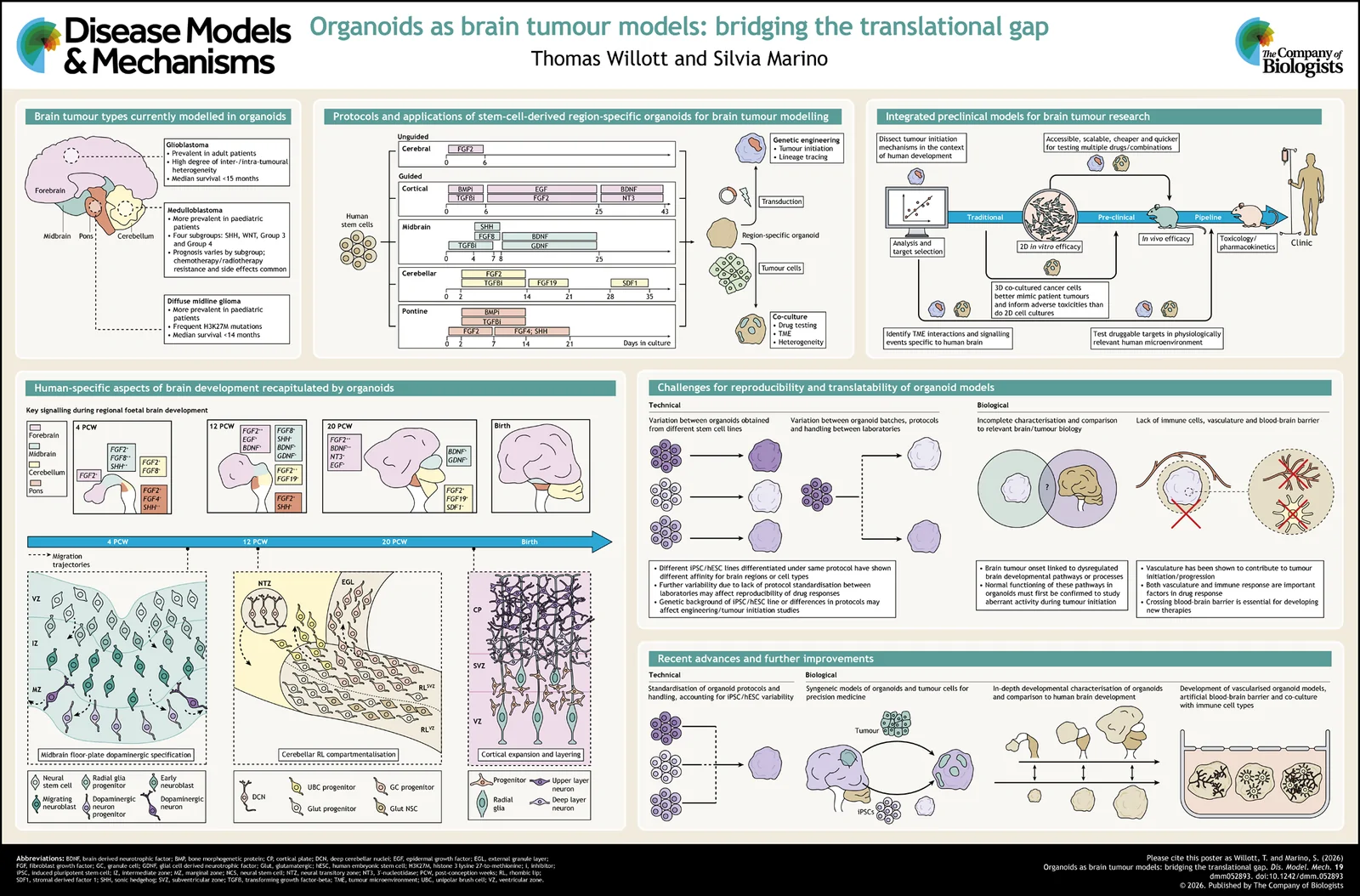
See supplementary information for a high-resolution version of the poster.

## INTRODUCTION

Despite decades of research, progress in developing effective therapies for both adult and paediatric primary malignant brain tumours (see poster, ‘Brain tumour types currently modelled in organoids’) has been frustratingly slow. Survival outcomes continue to be poor (Louis et al., 2021), notably for glioblastoma, the most prevalent adult brain tumour, as well as for the more aggressive subgroups of medulloblastoma and diffuse midline glioma. A key contributing factor to this limited progress is the inadequacy of pre-clinical models, which often fail to faithfully recapitulate the biological complexity and heterogeneity of human brain tumours.

Brain tumour researchers employ a range of models, including *in vitro*, *in vivo*, *ex vivo*, *in silico* and hybrid systems (Akter et al., 2021). The first animal models of brain malignancies generated tumours via carcinogenic exposure in rat or mouse brains, thereby causing spontaneous formation of various central nervous system (CNS) tumour types (Lantos, 1986). Since then, the development of patient-derived xenografts has enabled the study of human cancer cells in an intact organism, while genetically engineered models in rodents or zebrafish perturbing driver genes and pathways have been instrumental in elucidating tumour biology (Akter et al., 2021). However, species-specific differences in brain development, immune composition and drug metabolism, can hamper the translational relevance of these models (Hermans and Hulleman, 2019). Recent large-scale comparative studies have highlighted evolutionary differences between mammalian brains, including cell types, developmental trajectories and transcriptional programmes that are either absent or substantially different in mice compared to humans (Bakken et al., 2021; Sepp et al., 2024). Bakken et al. (2021) showed that the human cortex contains transcriptionally specialised neuronal populations associated with more complex cognitive processing, which are not fully present in mice. Further, Sepp et al. (2024) demonstrated that human cerebellar development is governed by species-specific regulatory programmes that drive prolonged expansion of human cerebellar progenitors and increased cellular complexity (Sepp et al., 2024). The differences between the human and mouse brains are not limited to normal development processes. Putative cells of origin for some brain tumours and their associated transcriptional states, including glioblastoma (Hamed et al., 2025; Thomas et al., 2008) and medulloblastoma (Smith et al., 2022; Hendrikse et al., 2022), have been identified as underdeveloped in mice. These findings highlight the limitations of current mouse models in dissecting the ontogeny of these brain tumours.

Conventional two-dimensional (2D) cell culture systems derived from primary human tumour tissue have enabled extensive molecular profiling and elucidation of regulatory mechanisms in brain tumours owing to their scalability and relatively straightforward manipulation, such as hijacking of stemness mechanisms in glioblastoma (Bulstrode et al., 2017) and epigenetic dysregulation in paediatric diffuse midline gliomas (Castel et al., 2018). However, establishing 2D cultures for some brain tumour types, including particular subgroups of medulloblastoma and isocitrate dehydrogenase-mutant gliomas, has proven difficult (Badodi et al., 2019). Moreover, monolayer cell cultures fail to recapitulate the three-dimensional (3D) architecture and microenvironment contributions (Greenwald et al., 2024), cellular heterogeneity (Neftel et al., 2019) and developmental origins (Wang et al., 2025; Kiang et al., 2025) of brain tumour growth and therapeutic response.

An alternative to mouse models and *in vitro* 2D cell culture, human *ex vivo* organotypic tumour slice cultures preserve native tumour architecture and microenvironmental components (Mann et al., 2025). However, their use is restricted by limited tissue availability, short culture windows up to a maximum of 6 weeks and an inability to model tumour initiation or long-term evolution (Steindl and Valiente, 2025). Another alternative is organoid models derived from brain tumour tissue, the applications of which to drug testing and understanding the biology of fully developed tumours have been comprehensively reviewed elsewhere (Mann et al., 2025). However, these models are not suitable for modelling tumour initiation and drug response in a healthy neurophysiological environment.

The advent of induced pluripotent stem cell (iPSC) technology (Takahashi et al., 2007; Yang et al., 2017) has transformed disease modelling, enabling the generation of self-organising, 3D brain organoids that recapitulate key aspects of human neurodevelopment, including neural stem cell induction, neuronal/glial lineage differentiation and architecture resembling structures of the developing brain (Mulder et al., 2023). These systems offer a unique opportunity to bridge the gap between basic *in vitro* models and patient tumours by combining human-specific developmental programmes and physiology with experimental tractability (see poster, ‘Human-specific aspects of brain development recapitulated by organoids’ and ‘Integrated preclinical models for brain tumour research’). Over the past decade, human embryonic stem cell (hESC)- or iPSC-derived brain organoids have been increasingly adopted in neuro-oncology (Alves et al., 2025), with the development of forebrain cerebral (Lancaster et al., 2013) and cerebellar organoids (Muguruma et al., 2015) facilitating initial studies of adult glioblastoma (Ogawa et al., 2018; Linkous et al., 2019) and paediatric medulloblastoma (Ballabio et al., 2020), respectively. The brain-region specificity of stem cell-derived organoids with cerebral/forebrain, midbrain and cerebellar identity provides compartmentally restricted physiological environments for the study of tumours typically occurring in these locations, which will be discussed in more detail in the following sections (see poster, ‘Human-specific aspects of brain development recapitulated by organoids’).

This At a Glance article focuses on how region-specific stem cell-derived brain organoids are being specifically leveraged to model adult and paediatric brain tumours, with particular emphasis on modelling strategies and developmental parallels. We discuss how these models have already yielded novel biological insights into cancer mechanisms, such as aberrant oncogene regulation (Wang et al., 2024), dysregulated developmental programmes (Willott et al., 2026) and tumour–microenvironment interactions (Mangena et al., 2025). We further critically evaluate their current limitations and highlight strategies to improve their clinical relevance and translatability.

## Stem cell-derived organoids to model region-specific aspects of human brain development

### Region-specific organoid protocols

A defining strength of brain organoids lies in their ability to recapitulate region-specific aspects of human brain development, thus providing physiologically relevant settings for region-specific brain tumours. The first unguided cerebral organoid protocol developed by Lancaster et al. (2013) relied on intrinsic self-patterning of pluripotent stem cells embedded in extracellular matrix scaffolds, producing heterogeneous lineages and structures resembling predominantly forebrain regions, although some midbrain, hindbrain and choroid plexus populations were also identified. These models capture broad cortical organisation, including the formation of ventricular zone-like structures containing polarised layers of neural stem and progenitor cells and differentiation of radial glia (Eichmüller and Knoblich, 2022). However, the reliance of unguided protocols on endogenous iPSC characteristics, which can vary between lines (discussed later in this article), led to differences in proportions of regional identity of the resultant cerebral organoids.

Subsequent studies, therefore, introduced guided differentiation strategies with selected growth factor and media constituents, mimicking patterning cues of the developing brain to generate more standardised, region-specific organoids (see poster, ‘Protocols and applications of stem-cell derived region-specific organoids for brain tumour modelling’). These protocols are based on the principle of sequential activation of key neurodevelopmental pathways driving region-specific cell lineage specification during organoid differentiation (see poster, ‘Key signalling during regional foetal brain development’ in ‘Human-specific aspects of brain development recapitulated by organoids'; and ‘Protocols and applications of stem-cell derived region-specific organoids for brain tumour modelling’).

Although unguided cerebral organoids show an affinity toward forebrain differentiation (Lancaster et al., 2013), it is only upon further refinement by Paşca et al. (2015), who employed dual SMAD inhibition and carefully timed growth factor exposure, that almost exclusively dorsal forebrain identities were achieved with reproducible populations of excitatory neurons and radial glia-like progenitors. These methods have since been further developed to improve radial glia cytoarchitecture (Rosebrock et al., 2022) or neuroepithelial cortical cell expansion (Pagliaro et al., 2023). Additional adaptations have also been made to cortical/forebrain organoid protocols, tailoring the cell identities and architecture to understand neurodegeneration (Shimada et al., 2022), connectivity (Choe et al., 2025) and eye development (Gabriel et al., 2023).

In contrast, cerebellar organoid protocols mimic early foetal signalling by the isthmic organiser, a key structure at the midbrain–hindbrain barrier governing its development, followed by growth factor induction of lineages derived from the cerebellar rhombic lip (a transient foetal structure that gives rise to multiple cerebellar glutamatergic lineages) and ventricular zone (Silva et al., 2020; Muguruma et al., 2015). Other cerebellar organoid models developed subsequently have employed alternative growth factor combinations, as well as retinoic acid, to more closely mimic early cerebellar morphogenesis (Atamian et al., 2024; Nayler et al., 2021).

Stem cell-derived organoids modelling regions of the brainstem have also been generated, with guided differentiation towards midbrain dopaminergic (Jo et al., 2016) or pontine (Bessler et al., 2026) regional identities. Midbrain organoids rely on early activation of WNT and sonic hedgehog (SHH) signalling to induce midbrain floor plate regional identity and dopaminergic neuron differentiation (Jo et al., 2016), which have since been further refined (Eura et al., 2020) or standardised to improve uniformity and scalability (Sarrafha et al., 2021). Most recently, guided protocols to generate pontine brainstem organoids were developed utilising fibroblast growth factor 4 (FGF4) signalling to drive glial differentiation characteristic of the pons region, with the organoids then utilised to interrogate the onset of paediatric diffuse midline gliomas (Bessler et al., 2026).

A key issue facing researchers deciding which region-specific organoid methodology to select for brain tumour modelling is the lack of side-by-side comparisons of organoids generated by these protocols with identical stem cells and handling procedures. Nevertheless, the selection of appropriate brain region-specific organoids for the tumour being modelled is paramount and has been extensively reviewed elsewhere (Mulder et al., 2023). This article will focus on the limitations common to all stem-cell-derived brain organoids for tumour modelling and how they may be addressed. Indeed, successful regional differentiation depends not only on protocol design but also on intrinsic properties of stem cell lines, including epigenetic landscape, donor variability and iPSC reprogramming method (Cahan and Daley, 2013). Differences in responsiveness to patterning cues can substantially alter cellular composition (Naas et al., 2024 preprint), maturation state and reproducibility (Bertucci et al., 2023 preprint), with direct implications for tumour modelling. As discussed later, these sources of variability are now starting to be addressed through further protocol standardisation and benchmarking efforts.

### Recapitulation of human neurodevelopment relevant to tumour biology

Cerebellar, cortical and brainstem organoids have the potential to significantly contribute to the dissection of the developmental origin and heterogeneity of medulloblastoma, glioblastoma and diffuse midline glioma, respectively. Understanding human neurodevelopment is an essential first step to clarifying how these tumours originate, progress and respond to therapy (see poster, ‘Human-specific aspects of brain development recapitulated by organoids’).

#### Cerebellar organoids

The rhombic lip is a transient structure of the foetal cerebellum responsible for the production of glutamatergic neurons, wherein the commitment of neural stem cells to glutamatergic progenitor cell fates, followed by their rapid expansion and maturation, is tied to their outward migration to other cerebellar compartments (Sepp et al., 2024). Recent studies comparing human cerebellar single-cell RNA-sequencing (scRNA-seq) atlases of foetal development and tumour transcriptional profiles have demonstrated that distinct rhombic lip progenitor populations correspond to specific medulloblastoma subgroups, including Group 3 and Group 4, serving as putative cells of origin for these tumours (Hendrikse et al., 2022; Smith et al., 2022; Visvanathan et al., 2024). Other work has also demonstrated that the spectrum of Group 3, Group 4 and intermediate medulloblastoma subtypes mirrors the transcriptional profiles of foetal cerebellar glutamatergic lineages as they mature (Williamson et al., 2022), further implicating dysregulated cerebellar development in medulloblastoma onset (Marino, 2005). It is, however, unclear how faithfully these progenitor states and trajectories are conserved in mice (Butts et al., 2024; Smith et al., 2022), providing a potential explanation for longstanding difficulties in modelling these subgroups in animal systems. Cerebellar organoids demonstrate structural organisation resembling the rhombic lip (Muguruma et al., 2015; Silva et al., 2020), in some cases even forming continuous polarised rhombic lip neuroepithelium (Muguruma et al., 2015), as well as glutamatergic progenitors with rhombic lip ventricular or subventricular zone compartment identities (Atamian et al., 2024). In medulloblastoma, our recent work has demonstrated the presence of putative Group 3 and Group 4 cells of origin at a matched developmental stage in cerebellar organoids (Willott et al., 2026). Together, these findings highlight the relevance of human-specific developmental systems for brain tumour modelling.

#### Cortical organoids

Cortical organoids recapitulate key features of human cerebral cortex development (Paşca et al., 2015), revealing the emergence and diversification of radial glia-like progenitor states, including outer radial glia populations that are largely unique to human corticogenesis, and play important roles in cortical expansion and neuronal lineage specification (Bhaduri et al., 2020a). Importantly, these progenitor states mirror transcriptional programmes observed in glioblastoma. Single-cell analyses have shown that glioblastoma exhibits extensive intra- and inter-tumoral heterogeneity, harbouring dynamic cellular states that resemble neuronal, astrocytic or oligodendrocyte progenitors, as well as mesenchymal cell states (Neftel et al., 2019). The presence of these cell types within cortical organoids, therefore, provides a physiologically relevant platform to investigate cell identity and state transitions. Furthermore, stem-like glioblastoma subpopulations are thought to contribute to therapeutic resistance and tumour recurrence through their capacity for self-renewal and phenotypic plasticity (Prager et al., 2020). Consequently, cortical organoids provide a promising avenue for further research to understand cell fates important in normal neurodevelopment and glioblastoma plasticity.

#### Brainstem organoids

Midbrain organoids were first shown to recapitulate key features of human midbrain development, including the generation of functional dopaminergic neurons (Jo et al., 2016). Dopaminergic signalling plays an essential role in regulating neuronal differentiation, maturation and circuit formation within the developing midbrain (Hegarty et al., 2013). Components of these signalling pathways are dysregulated in diffuse midline gliomas and have emerged as potential therapeutic targets in tumours harbouring histone 3 lysine 27-to-methionine (H3K27M) mutations, with dopamine receptor antagonists currently in clinical trials (Arrillaga-Romany et al., 2024). More recently, Bessler et al. (2026) generated brainstem organoids modelling the cellular and molecular programmes present within the developing pons, the most common anatomical site of diffuse midline glioma (Vitanza and Monje, 2019). They demonstrated that these organoids undergo enhanced pontine glial differentiation, generating larger and more faithful populations of these cells than other brainstem organoid protocols, thereby better recapitulating the developmental cell states required for tumour formation (Bessler et al., 2026).

Together, these examples highlight how organoids capture human developmental contexts that are mechanistically informative for tumour initiation, progression and treatment.

## Current applications of organoids to brain tumour modelling

### Co-culture with patient-derived tumour cells

One widely adopted strategy for modelling brain tumours in organoids involves the seeding of patient-derived tumour cells into pre-formed brain organoids. In these co-culture systems, tumour cells are added onto organoids, where they invade, proliferate and interact with a structured, human neural microenvironment (Alves et al., 2025). This allows the study of tumour progression, microenvironment interactions and drug response in a 3D, physiologically relevant context, mimicking cell interactions and signalling, which have been shown to significantly contribute to brain tumour biology (Quail and Joyce, 2017). Importantly, for drug-testing applications, tumour-organoid co-culture systems enable the simultaneous assessment of drug effects on both neoplastic and non-neoplastic cell populations, thereby providing insight into both on- and off-target toxicities.

#### Tumour biology

Linkous et al. (2019) first performed co-culture of primary patient-derived glioblastoma stem cells with cerebral organoids (GLICOs) obtained from H1, H6 or H9 human stem cell lines, with all tested tumour cell lines demonstrating successful engraftment in the cerebral organoid. They further demonstrated that glioblastoma cells invading cerebral organoids recapitulate key features of patient tumours, including diffuse infiltration, transcriptional heterogeneity and therapy resistance (Linkous et al., 2019). Importantly, glioblastoma cells co-cultured with organoids exhibited increased resistance to standard-of-care chemotherapy compared with matched cell lines cultured and treated in two dimensions, better reflecting the poor treatment response and recurrence broadly observed in patients (Linkous et al., 2019). This underscores the influence of tissue architecture and cell–cell interactions on tumour drug responses. Subsequently, GLICOs have been used to investigate resistance mechanisms commonly found in glioblastoma tumours (Russo et al., 2026). Multiple groups have employed this model to study the dynamics of glioblastoma invasion, including its effects on healthy neural populations at different developmental stages (Krieger et al., 2020), to develop scalable invasion assays (Goranci-Buzhala et al., 2020), to characterise preferential invasiveness in different forebrain regions (Fan et al., 2024), and to understand the invasiveness and plasticity of glioblastoma stem cell subtypes (Bhaduri et al., 2020b). Remarkably, Mangena et al. (2025) further showed that patient-derived glioblastoma cells co-cultured with cortical organoids retained inter- and intra-tumour heterogeneity over extended culture periods, up to 6 months, including the preservation of pro-neuronal transcriptional programmes, which are associated with glioblastoma infiltration (Mangena et al., 2025). They also demonstrated the transfer of tumour mRNA to surrounding non-malignant cells via extracellular vesicles, promoting cellular crosstalk, providing a system to dissect the integration of glioblastoma into neural circuits, which has been previously linked to poorer prognosis (Krishna et al., 2023). Cerebral organoid and tumour co-culture systems have also been shown to faithfully recapitulate other important aspects of tumour biology and treatment, including hypoxia-driven pro-tumour signalling (Nicholson et al., 2024) and comparable response of organoid tissue to radiotherapy-induced neuroinflammation (Millner et al., 2025).

Subsequent studies have extended this approach to paediatric brain tumours and relevant region-specific organoids. van Essen et al. (2024b) co-cultured SHH medulloblastoma cell lines with cerebellar organoids and subsequently performed single-cell profiling. The results revealed that tumour cells grown in co-culture exhibited transcriptomic heterogeneity, more closely resembling the same cells maintained as xenografts in mice rather than cells grown in 2D culture (van Essen et al., 2024b). In keeping with these results, we demonstrated that co-culture of cerebellar organoids with Group 3 medulloblastoma cells better phenocopy patient tumours than do matched 2D medulloblastoma cell cultures. These models also recapitulate therapeutic responses to targeted therapies seen in mice as well as patient responses to standard-of-care chemotherapy, highlighting the utility of co-culture models for further drug development efforts (Willott et al., 2026). Importantly, the physiological environment provided by 3D organoid co-cultures may enable expansion and further characterisation of those tumours, such as Group 4 medulloblastoma, isocitrate dehydrogenase-mutant gliomas and other low-grade gliomas, which cannot be cultured in two dimensions (Badodi et al., 2019).

#### Tumour microenvironment

Co-culture systems are also powerful for studying the tumour microenvironment (TME), encompassing CNS tissue, vessels, inflammatory cells and extracellular matrix (Khan et al., 2025). Current co-culture models are best suited for dissecting interactions between the tumour and various non-neoplastic neural cell types, with organoids harbouring committed neuronal, astrocyte and oligodendrocyte lineages. Astrocytes have been shown to promote invasion and progression in glioblastoma, and neuronal signalling also promotes tumour proliferation (Quail and Joyce, 2017). Linkous et al. (2019) demonstrated that glioblastoma cells actively remodel the surrounding neural tissue in cerebral organoids, forming microtubule networks with neurons, as well as preserving parental tumour receptor tyrosine kinase signalling via phosphorylation of epidermal growth factor receptor (EGFR) and ephrin receptors. Pine et al. (2020) further demonstrated that the healthy cerebral organoid microenvironment was essential for maintaining the heterogeneity of patient glioblastoma tumours upon *in vitro* culture, as well as for utilising co-culture models to dissect epigenetic regulation of glioblastoma plasticity. These findings support the growing evidence that functional interactions between tumour cells and neural cell networks contribute to glioblastoma biology.

The medulloblastoma TME is less well characterised, although several studies have identified astrocyte-mediated regulation of stemness contributing to tumour progression and relapse, while interactions with neurons are less well understood, highlighting the need for additional models (van Bree and Wilhelm, 2022). Gene expression profiling of aggressive medulloblastoma tumours (including Group 3) has determined the upregulation of the transforming growth factor-beta (TGFβ) pathway to be associated with poorer prognosis (Aref et al., 2013; Northcott et al., 2011). These results correlate with the TGFβ pathway activation known to be mediated by tumour microenvironment factors in other cancers (Pickup et al., 2013). Our scRNA-seq profiling of human Group 3 medulloblastoma cells and cerebellar organoid co-cultures predicted interactions between tumour and neural cells mediated by the TGFβ pathway. This prediction was confirmed by subsequent pharmacological inhibition of TGFβ receptor 3, which resulted in impeded tumour cell growth within the co-culture system (Willott et al., 2026). SHH medulloblastoma cells in co-culture with cerebellar organoids have also demonstrated expression of stem cell programmes and the neuronal differentiation 1 (*NEUROD1*) gene, both permitting a cycling cell state seen in tumours that was not found in matched 2D cultured tumour cells (van Essen et al., 2024b). This further highlights the role of the TME in brain tumour biology, which can be recapitulated in co-culture models.

These models are, however, not without limitations. For example, they are inherently not suitable for studying tumour initiation, as cells are isolated from advanced tumours that have undergone multiple series of mutations and cancer evolution dynamics over a prolonged period of time. Although 3D co-cultures of organoids and tumour cells are an attractive option for testing novel anti-cancer drugs and quantifying toxicities on non-neoplastic cells to inform potential adverse effects, current methodologies limit the scalability of these models for large high-throughput drug screening owing to the commonly used manual handling protocols (Mulder et al., 2023). Moreover, the lack of TME components, such as immune cell types and vascularisation, which have been shown to affect therapeutic response (Khan et al., 2025; Quail and Joyce, 2017), should also be considered in future studies.

### Genetically engineered organoids

Genetic manipulation via CRISPR-Cas9-mediated genome editing or viral gene delivery has been used to introduce oncogenic mutations or pathway perturbations into developing organoids to model tumour onset. This technique allows the dissection of specific gene contributions or combinations of driver events to brain tumour initiation. It also benefits from the tractability of organoid models, which enables the analysis of cells at multiple time points during tumorigenesis, as well as easier tracking and manipulation than in *in vivo* genetically engineered mouse models.

#### Cerebral organoids

Glioblastoma tumours frequently harbour inactivating mutations in a wide range of tumour suppressor genes [including tumor protein p53 (*TP53*), phosphatase and tensin homolog (*PTEN*), cyclin dependent kinase inhibitor 2A/B (*CDKN2A/B*), ATRX chromatin remodeler (*ATRX*), RB transcriptional corepressor 1 (*RB1*) and neurofibromin 1 (*NF1*)] and gain-of-function mutations [including those in *EGFR*, epidermal growth factor receptor variant III (*EGFRvIII*), platelet derived growth factor receptor alpha (*PDGFRA*) and cyclin dependent kinase 4 (*CDK4*)] (Sturm et al., 2014). Genetically engineered cerebral organoids have been used to further understand the roles of these genes' dysregulation in glioblastoma initiation. Ogawa et al. (2018) first employed CRISPR-Cas9 to knock out *TP53* activity, which, in combination with increased RAS/MAPK signalling, also commonly upregulated in individuals with glioblastoma (Feldkamp et al., 1999), gave rise to proliferative and invasive tumours. Subsequently, Bian et al. (2018) used CRISPR-Cas9 and transposon-mediated cerebral organoid engineering to investigate common glioblastoma mutations via combinations of tumour suppressor gene knockout (^–/–^) and oncogene overexpression (^OE^) (Bian et al., 2018). Interestingly, they found that only specific engineered mutation combinations (*CDKN2A*^–/–^/*CDKN2B*^–/–^/*EGFR*^OE^/*EGFRvIII*^OE^; *NF1*^–/–^/*PTEN*^–/–^/*TP53*^–/–^ and *EGFRvIII*^OE^/*CDKN2A*^–/–^/*PTEN*^–/–^) could give rise to tumours, with varying expression of stem and glial markers in engineered tumour-bearing organoids reflecting glioblastoma heterogeneity. Similar approaches focusing on commonly mutated glioblastoma tumour suppressors followed by extensive multi-omic characterisation have demonstrated the spatial dysregulation caused by specific mutation combinations (Ishahak et al., 2025) and identified potential drug targets involved in lipid metabolism (Wang et al., 2024). Genetically engineered cerebral organoids have also been used to identify new aspects of glioblastoma epigenetic regulation. Overexpression of mesenchyme homeobox 2 (*MEOX2*), encoding a transcription factor upregulated in glioblastoma (Tachon et al., 2021), in cerebral organoids led to glioblastoma formation through activation of aberrant ERK signalling via epigenetic reprogramming, identifying novel and potentially druggable regulatory mechanisms (Schönrock et al., 2022). Similarly, cerebral organoids have been leveraged to characterise switches between astrocytic and neuronal progenitor cell fates mediated by the DNA methyltransferase *METTL7B* (also known as *TMT1B*) during early lineage specification in glioblastoma, mirroring processes seen in patient tumours that promote invasiveness (Constantinou et al., 2024). Together, these data demonstrate the utility of genetically engineered brain organoids to dissect the mutational landscape of tumours.

#### Cerebellar organoids

Genetic engineering approaches have also been used for modelling medulloblastoma in cerebellar organoids. Initially, Group 3 medulloblastoma was modelled via electroporation of constructs conferring overexpression of orthodenticle homeobox 2 (*OTX2*) or growth factor independent 1 transcriptional repressor (*GFI1*) and MYC proto-oncogene, bHLH transcription factor (*MYC*) oncogenes highly expressed in these tumours (Cavalli et al., 2017) into bulk cerebellar organoids. Upon further differentiation, engineered organoids developed clusters of highly proliferative cells that formed tumours resembling medulloblastoma upon orthotopic xenograft (Ballabio et al., 2020). Similarly, SHH medulloblastoma was modelled via CRISPR-Cas9 mediated knockdown of the SHH receptor patched 1 (*PTCH1*) in iPSCs prior to differentiation, with the resultant organoids demonstrating dysregulated development and transcriptional resemblance to patient tumours (van Essen et al., 2024a). These studies highlight the utility of genetically engineered cerebellar organoids for medulloblastoma modelling, particularly enabling investigation of the formation of different subgroups.

#### Brainstem organoids

Diffuse midline gliomas occurring in the pons frequently harbour H3K27M mutations, leading to an aggressive tumour phenotype (Louis et al., 2021). To investigate the role of the H3.3K27M mutation in gliomagenesis, Bessler et al. (2026) electroporated pontine brainstem organoids with plasmids conferring the mutation, as well as *TP53* and *PDFDRA* alterations, observing invasive outgrowth of mutant cells tied to dysregulated pontine lineage specification and methylation profiles clustering with patient diffuse midline glioma tumours (Bessler et al., 2026). This further highlights the utility of stem cell-derived organoids for dissecting tumours linked to aberrant developmental processes, prevalent in paediatric patients.

Genetically engineered organoids are uniquely suited for studying tumour initiation, lineage hierarchy and early evolutionary trajectories owing to the developmentally faithful dynamic cellular context of early stem and committed progenitor states progressing through differentiation. Recently, numerous parallels between tumour initiation and normal brain development have emerged from human genomic and transcriptomic studies, including putative cells of origin, the dysregulated development of which is thought to give rise to tumours (Hendrikse et al., 2022; Smith et al., 2022; Hamed et al., 2025; Mathur et al., 2024; Wang et al., 2025). Genetically engineered stem cell-derived organoids, therefore, present a valuable system to dissect neoplastic ontogeny. However, current models lack the full genetic complexity of patient tumours, in particular chromosomal instability and heritable epigenetic states (Sturm et al., 2014), and may oversimplify tumour evolution by relying on a limited set of engineered alterations.

## Refinement of models and future avenues to enhance their translatability

### Standardisation and iPSC variability

A major challenge in organoid-based modelling is variability arising from differences in stem cell lines, differentiation protocols and culture conditions (see poster, ‘Challenges for reproducibility and translatability of organoid models’) (Eichmüller and Knoblich, 2022; Mulder et al., 2023). Recent comparisons of organoid differentiation protocols for cortical (Bertucci et al., 2023 preprint) or various forebrain/midbrain (Naas et al., 2024 preprint) regions have highlighted the extent of iPSC-driven variability due to donor genetics and epigenetic state, demonstrating that such variability can significantly influence organoid composition and regional identity. Most cerebral organoid-based glioblastoma modelling studies to date have utilised the widely available H1 or H9 hESC lines (Correia et al., 2025), with Naas et al. (2024 preprint) demonstrating varying responses to forebrain protocol differentiation cues of these cells, as well as other iPSC lines. The authors induced organoid differentiation from different stem cell lines with different protocols and systematically analysed these comparatively with scRNA-seq profiling followed by neighbourhood analysis to quantify the extent to which the pattern of cell groupings was influenced by the starting stem cell line. This approach allowed them to score organoids and determine whether cell fates were predominantly driven by intrinsic stem cell properties or response to patterning cues. Importantly, they found considerable variability between cell lineages, organoid protocols and stem cell lines, indicating that different lines demonstrate different responses to patterning cues and highlighting the need for further standardisation (Naas et al., 2024 preprint).

Azzarelli and colleagues optimised co-culture for cerebral organoids derived from three iPSC lines with five glioblastoma stem cell lines using defined media and growth conditions, focusing on the generation of viable, reproducible co-cultures modelling parental glioblastoma tumour phenotypes (Azzarelli et al., 2021). Although all glioblastoma cell lines tested could be co-cultured, they found that only one iPSC line generated organoids with desirable reproducibility for subsequent co-cultures between batches. Along similar lines, Atamian et al. (2024) showed that cerebellar organoids cultured under identical conditions displayed varying cell types and fidelity depending on the iPSC/hESC line used, again most likely reflecting different genetic backgrounds of stem cells obtained from different patients (Ortmann et al., 2020). Indeed, it remains unclear whether the stemness of the hESC/iPSC line utilised is an important factor to consider, particularly given the development of reprogramming protocols generating expanded potential stem cells (EPSCs), which exhibit totipotent characteristics compared with pluripotency in iPSCs (Yang et al., 2017). In our hands, EPSCs were capable of differentiating into cerebral or cerebellar organoids with high fidelity, with the same starting EPSC lines able to properly differentiate into cell types specific to each of these regions following each protocol (Vinel et al., 2021; Constantinou et al., 2024; Willott et al., 2026). However, a systematic comparison with matched syngeneic reprogrammed iPSCs would be needed to identify true differences driven by stemness and reprogramming methodology. Variability within batches of organoids remains an important factor to consider in both stem cell lines and protocols for organoid-based disease modelling (Quadrato and Arlotta, 2017).

In summary, inter-line variability is a current limitation of organoid-based modelling as it can rival or exceed the disease-associated effects being modelled. This is particularly important in tumour initiation, during which subtle differences in developmental state can profoundly influence tumour behaviour. For TME investigations, receptor–ligand modelling compares single-cell transcriptional profiles, meaning that predictions must be based on gene expression levels caused by differentiation into TME-relevant organoid cell types, rather than the starting stem cell line used. To improve accessibility and flexibility in study design and downstream applications, researchers should address stem-cell variability by optimising protocol reproducibility, rather than relying on the selection of superior lines (Bertucci et al., 2023 preprint), allowing broader adoption and cross-study comparability.

### Syngeneic tumour-organoid systems

Insights into brain cancer biology have been gained by the molecular profiling and comparison of tumours between patients, as well as, more recently, by comparing spatial datasets or developmentally matched normal brain samples (see poster, ‘Recent advances and further improvements’) (Porter et al., 2023; Kuehn et al., 2024). These studies have reinforced the appreciation of the high degree of inter- and intra-tumoral heterogeneity (Cavalli et al., 2017; Clarke et al., 2020; Badodi et al., 2017), particularly in glioblastoma (Neftel et al., 2019), which strongly advocates for treatments tailored to individual patients and molecularly defined subgroups of patients. To begin to model this important aspect of glioblastoma biology, syngeneic systems have been developed in which tumour cells and organoids are derived from the same individual. Vinel et al. (2021) demonstrated that glioblastoma cells co-cultured with cerebral organoids derived from syngeneic non-neoplastic EPSC maintain inter-patient differences in invasion and drug sensitivity, enabling personalised therapeutic prediction and evaluation (Vinel et al., 2021). Interestingly, co-culture of patient-matched glioblastoma and immune cells with non-syngeneic cortical organoids has also recently been described, allowing the characterisation of patient-specific immunotherapy responses (Baisiwala et al., 2026). Integrating these two methodologies to obtain syngeneic models of non-neoplastic organoid, tumour and immune cell co-cultures will be a promising future avenue for precision oncology.

### Deep molecular benchmarking and engineering cells of origin

Increasingly, organoid models are being benchmarked against human developmental datasets at both transcriptomic and epigenetic levels to assess how accurately they recapitulate developmental processes (Luo et al., 2016; Willott et al., 2026; Kanton et al., 2019; Camp et al., 2015; Amiri et al., 2018; He et al., 2024). These comparisons with foetal brain atlases have demonstrated the extent of transcriptomic and epigenomic molecular similarity achieved by organoid protocols, providing important validation of their utility as models of human neurodevelopment. As organoids are shown to faithfully reproduce key developmental cell states and trajectories, they also offer the opportunity to generate and experimentally manipulate putative tumour cells of origin within a physiologically relevant developmental context. Cells of origin are thought to be particularly important in brain tumours, given their onset during foetal development (Kiang et al., 2025; Wang et al., 2025) or hijacking of developmental processes (Mathur et al., 2024), although how and why these specific cell lineages or states are susceptible to tumour initiation is not known.

Our recent work characterised both the transcriptome and epigenome of cerebellar organoids relative to the human foetal cerebellum, identifying populations enriched for putative medulloblastoma cell-of-origin signatures at developmental stages corresponding to tumour initiation during foetal cerebellar morphogenesis (Willott et al., 2026). Targeted overexpression of the *MYC* oncogene, frequently amplified in aggressive medulloblastomas (Cavalli et al., 2017), within these organoid-derived cells of origin demonstrated their susceptibility to neoplastic transformation, generating medulloblastoma tumours following orthotopic transplantation in mice (Willott et al., 2026). Molecular profiling and comparison to patient tumours further highlighted the improved fidelity of these lineage-targeted models relative to previous bulk genetically engineered cerebellar organoid models (Ballabio et al., 2020).

Lineage-restricted genetic engineering approaches, in which oncogenic alterations are selectively introduced into defined cell populations rather than across the whole organoid, have also been used to characterise gliomagenesis, specifically targeting neural stem cell populations thought to function as glioblastoma cells of origin (Mathur et al., 2024). Lineage-restricted knockdown of the commonly mutated tumour suppressor genes *TP53*, *PTEN* and *NF1* in nestin (*NES*)-positive neural stem cells in cerebral organoids generated cells with a mutational landscape and phenotypes consistent with the pro-neural glioblastoma subtype (Singh et al., 2023).

These studies demonstrate the importance of extensive molecular characterisation and targeting relevant cell populations to accurately model tumour initiation, particularly for those brain tumours with an ontogeny linked to dysregulated developmental processes.

### Enhancing generalisability and scalability of models

The choice between co-culture and genetic engineering approaches depends strongly on the biological question being addressed. Co-culture systems are favoured for modelling tumour invasion, microenvironmental interactions and drug response, particularly when using primary patient-matched tumour cells. In contrast, genetically engineered organoids are better suited for mechanistic studies of tumour initiation and the functional consequences of specific genetic alterations. Although most engineered models have focused on glioblastoma and medulloblastoma, emerging studies suggest that these approaches could be extended to other brain tumour types and associated cellular lineages including ependymoma (Gojo et al., 2020), diffuse intrinsic pontine gliomas (Nagaraja et al., 2017) and other high-grade brain tumours (Wang et al., 2025). Co-culture of other region-specific tumour cell lines derived from different subtypes of high-grade gliomas, including ependymoma and neuroblastoma (Xu et al., 2015), with organoids modelling relevant brain regions also represents an avenue for further exploration.

Despite recent advances, both approaches still face limitations in scalability, impeding their translatability. As well as efforts from the community to address variability arising from protocol and stem cell line differences, the development of higher-throughput protocols is critical for wider adoption. One such example is methods for culturing stem cell-derived organoids in bioreactors (Silva et al., 2021; Ramani et al., 2024). However, following organoid generation by these protocols, the manual process of co-culturing tumour cells and analysing therapeutic response remains a limiting factor for drug screening. Recent studies demonstrating the use of robotics and automated platforms for organoid handling (Boussaad et al., 2021), imaging and phenotypic analysis (Bozal et al., 2024) have shown that these methods can substantially increase throughput. Boussaad et al. (2021) demonstrated the possibility of lowering costs of organoid generation by replacing manual iPSC culture and midbrain organoid generation with automation. Meanwhile, Bozal et al. (2024) integrated high-content imaging as a phenotypic readout with biochemical viability assays to enable robust assessment of response to chemotherapy and kinase inhibitors in colorectal cancer organoids. Integration of automated cell cultures and liquid-handling robotics with protocols for brain organoid generation and co-culturing with brain tumour cells has the potential to improve their suitability for large-scale drug screening and repurposing studies, thus increasing the likelihood that these findings can be translated for clinical practice. A further opportunity to improve the scalability and flexibility of these systems for drug testing applications is to extend the culture window of brain tumour organoid models, permitting further experiments on tumour evolution and therapy resistance mechanisms. This may be achieved through biophysical engineering approaches (Fan et al., 2025) or derivation of organoids from native foetal brain stem cells, which can expand in culture longer than their iPSC-derived counterparts (Hendriks et al., 2024), although the applicability of the latter approach is limited by availability and ethical constraints.

### Incorporating additional tumour-relevant features

Another key future direction in organoid-based brain tumour modelling is to incorporate additional features critical for tumour biology and therapeutic responses (see poster, ‘Challenges for reproducibility and translatability of organoid models’). Vascularisation remains an important consideration when investigating tumour heterogeneity, TME interactions and drug responses owing to its role in tumour cell signalling, plasticity and drug delivery (Guyon et al., 2021). Therefore, novel approaches ranging from endothelial co-culture (Shi et al., 2020) to genetic induction of vascular-like networks in organoids (Cakir et al., 2019; Sato et al., 2023) are being developed. Similarly, the inclusion of immune components such as microglia and peripheral immune cells is beginning to enable studies of immune interactions in organoid models (Fagiani et al., 2024; Gerasimova et al., 2025). For example, the immunosuppressive TME was shown to contribute to disease progression and therapy response in glioblastoma (Chen and Hambardzumyan, 2018). In accordance with this, co-culture of patient-matched immune and glioblastoma cells in cortical organoids enabled the characterisation of pembrolizumab-induced immunotherapy response, with patients displaying varied T-cell clonal expansion following treatment (Baisiwala et al., 2026). Interestingly, Peng et al. (2025) established the co-culture of cerebral organoids with a range of primary and metastatic brain tumour explants containing tumour, vasculature and immune components (Peng et al., 2025). They showed successful invasion and proliferation of glioblastoma tumour cells within the co-culture, while preserving the immune microenvironment and microvasculature elements and recapitulating individual patient responses to temozolomide standard-of-care chemotherapy (Peng et al., 2025). This approach presents unique advantages for personalised treatment, given that it preserves the patients' tumour structure and TME.

Modelling the blood–brain barrier (BBB) represents another important goal, particularly for drug delivery studies. Organoid-based BBB systems offer new opportunities to begin to incorporate this layer of complexity in tumour and organoid pharmacokinetics and toxicity in a human-relevant context (Dao et al., 2024; Bergmann et al., 2018; Ahn et al., 2021). However, the still incomplete knowledge of BBB-crossing mechanisms presents a hurdle to further model advancement.

By capturing human-specific developmental programmes, regional identities and cellular interactions, stem cell-derived brain organoids address fundamental limitations of traditional models and offer new avenues for mechanistic studies and therapeutic development. Although significant challenges remain, particularly in standardisation, scalability for high-throughput drug screening and incorporation of additional tumour-relevant features, stem cell-derived brain organoid tumour models are a valuable addition to the range of neuro-oncology modelling tools, contributing to closing the longstanding gap between *in vitro* models and patients.

## Poster

Poster
